# Effects of Different Fattening Periods on Slaughtering Performance, Meat Quality, and Muscle Histochemical Characteristics of Yanbian Cattle

**DOI:** 10.3390/ani16121846

**Published:** 2026-06-15

**Authors:** Depeng Sun, Yuankuo Sun, Zhen Liu, Jinliang Quan, Baide Mu, Chunxiang Piao, Guanhao Li

**Affiliations:** 1 College of Agriculture, Yanbian University, Yanji 133002, China; 2022001108@ybu.edu.cn (D.S.); 2056074133k@gmail.com (Y.S.); qq1684285293@163.com (Z.L.); ybdxqjl98@163.com (J.Q.); mubaide@ybu.edu.cn (B.M.); cxpiao@ybu.edu.cn (C.P.); 2 Key Innovation Laboratory for Deep and Intensive Processing of Yanbian High Quality Beef, Ministry of Agriculture and Rural Affairs, Yanji 133002, China

**Keywords:** fattening endpoint, histochemical characteristics, meat quality, slaughtering performance, Yanbian cattle

## Abstract

This study aimed to elucidate the effects of different fattening stages on slaughtering performance, meat quality, muscle fiber characteristics, connective tissue properties, and intramuscular fat deposition in Yanbian cattle. A total of 40 eighteen-month-old castrated male Yanbian cattle were raised together under identical conditions. At 24, 28, 32, and 36 months of age, 10 animals were randomly selected and slaughtered. Increased fattening periods, slaughter performance of Yanbian cattle improved steadily, carcass weight and meat weight increased significantly (*p* < 0.05). Dressing percentage and lean meat percentage reached their maxima at 32 months, which were 60.09% and 46.96% respectively. Cooking loss and centrifugal loss decreased with fattening time (*p* < 0.05), with the 32- and 36-month-old groups showing the best water-holding capacity. Intramuscular fat content increased significantly during fattening (*p* < 0.05), with no significant difference between the 32- and 36-month-old groups (*p* > 0.05). However, Warner–Bratzler shear force (WBSF) was significantly higher at 32 and 36 months than at 24 and 28 months (*p* < 0.05), indicating reduced tenderness with prolonged fattening. The endomysial collagen network at 32 months maintained a compact yet filamentous structure, whereas by 36 months it had transformed into a dense sheet-like configuration. In conclusion, fattening to 32 months achieves a compromise among marbling, collagen maturity, water-holding capacity, slaughtering efficiency, and the onset of increased toughness. Therefore, 32 months is recommended as the fattening endpoint when balancing meat production and quality traits.

## Simple Summary

In practical production, the selection of the fattening endpoint directly affects the growth performance, slaughter profitability, and meat quality of cattle. Indiscriminately prolonging the fattening period, with an excessive focus on quantitative traits such as fat deposition and meat yield while neglecting the influence of muscle fibers and intramuscular connective tissue on meat quality, may lead to an increase in carcass size but coarser muscle fibers, thereby compromising tenderness and juiciness. Yanbian cattle still lack systematic and scientific foundations regarding fattening regimes, slaughter age, and the determination of the optimal fattening endpoint. Therefore, this study aimed to systematically evaluate the effects of different fattening stages on the comprehensive performance of Yanbian cattle by integrating slaughtering performance, meat quality, and histochemical characteristics. This evaluation provides a basis for determining the optimal fattening endpoint and offers actionable insights for the beef cattle industry to enhance product consistency.

## 1. Introduction

Yanbian cattle, as one of the five major indigenous yellow cattle breeds in China, are renowned for their exceptional meat quality and unique flavor. Originating from the forested regions of the Changbai Mountains, they have been listed as a national geographical indication protected product [1]. However, within the context of the global high-end beef market, compared with the established grading system of Japanese Wagyu [2] and the high uniformity and standardized production of Australian Angus beef [3], Yanbian cattle still lack systematic and scientific foundations regarding fattening regimes, slaughter age, and the determination of the optimal fattening endpoint.

Meat tenderness is not only a primary quality indicator but also a core economic trait determining its market price. It is primarily governed by a combination of factors, including muscle fiber characteristics, sarcomere length, the content and degree of cross-linking of intramuscular connective tissue, and intramuscular fat content [4]. A smaller fiber diameter indicates more delicate partitioning of the perimysium, resulting in a finer overall texture of the muscle bundles, whereas larger muscle fibers correspond to greater shear force resistance, directly leading to reduced tenderness [5]. The influence of muscle fiber type on tenderness also extends into the dynamic biochemical processes of postmortem aging. Gagaoua et al. [6] demonstrated that muscles composed of a high proportion of type II fast-twitch fibers are more prone to proteolysis during the early postmortem period compared to muscles predominantly composed of type I fibers [7]. This phenomenon is attributed to the higher glycogen reserves and unique enzymatic properties of type II fibers [8]. These properties collectively influence the rate and extent of pH decline, indirectly modulate the activity of endogenous proteases, and ultimately determine the development of tenderness.

The “myofibrillar toughness” of beef is determined by myofibrils, whereas the “background toughness” is determined by connective tissue. This background toughness is primarily dictated by the collagen content and the degree of cross-linking [9]. Thermally stable, non-reducible mature trivalent collagen cross-links are the main factors determining the influence of connective tissue on meat tenderness [10]. Intramuscular fat, commonly referred to as marbling, is predominantly deposited within the perimysium of the intramuscular connective tissue [11,12], and its contribution to tenderness is manifested through a physical “dilution effect”. However, the regulatory effect of adipocytes on connective tissue is not static. As animals age, the increasing volume of adipocytes induces remodeling of the endomysium, leading to the formation of structurally diverse trivalent cross-links [13]. Furthermore, the effect of intramuscular fat levels on meat quality exhibits a threshold effect. When intramuscular fat content is below 8%, it primarily improves eating quality by enhancing flavor and juiciness. When fat levels exceed 8%, excessive fat deposition instead disrupts the structural integrity of the intramuscular connective tissue, resulting in increased shear force values [14]. Therefore, a precise balance must be sought between prolonging the fattening period to pursue marbling and timely slaughter to control background toughness.

In practical production, the selection of the fattening endpoint directly affects the growth performance, slaughter profitability, and meat quality of cattle. Indiscriminately prolonging the fattening period, with an excessive focus on quantitative traits such as fat deposition and meat yield while neglecting the influence of muscle fibers and intramuscular connective tissue on meat quality, may lead to an increase in carcass size but coarser muscle fibers, thereby compromising tenderness and juiciness [15]. Therefore, precisely defining the fattening endpoint that optimizes the meat quality of Yanbian cattle has become a critical parameter that urgently needs to be determined for production applications.

Currently, research on the fattening endpoint that relies solely on slaughtering performance indicators is insufficient to comprehensively capture the patterns of meat quality variation, let alone to elucidate the dynamic histochemical characteristics of muscle and adipose tissues. The properties of muscle fibers and intramuscular connective tissue, as well as the deposition patterns of intramuscular fat, constitute the microscopic basis determining meat tenderness and juiciness. Therefore, this study aimed to systematically evaluate whether different fattening stages affect slaughter performance, meat quality, intramuscular fat deposition, muscle fiber traits, and connective tissue characteristics in Yanbian cattle. The novelty of this study lies in its multi-parameter integration and identification of 32 months as a compromise endpoint between improved marbling and the onset of collagen-mediated toughening. These findings provide a scientific basis for determining the optimal fattening endpoint in Yanbian cattle production.

## 2. Materials and Methods

### 2.1. Selection and Feeding Management of Yanbian Cattle

A total of 40 eighteen-month-old castrated male Yanbian cattle (initial body weight: 395.88 ± 32.16 kg), sourced from Mule Animal Husbandry Co., Ltd. in Longjing City, China, were selected for this experiment. All 40 cattle were randomly allocated to slaughter age groups (24, 28, 32, 36 months; n = 10) using a random number table. Allocation was stratified by pen to ensure that each of the 8 pens (5 animals/pen; pen dimensions: 5.0 m × 8.0 m) contributed 1–2 animals to each slaughter age. All pens were managed identically in terms of feeding, watering, and environmental conditions. A total mixed ration feeding regimen was adopted, with feeding ad libitum twice daily (at 4:00 a.m. and 3:00 p.m.), and free access to water. The basal diet composition and nutritional levels of the fattening cattle are presented in Table 1.

### 2.2. Animal Grouping and Sample Collection

The experimental cattle were fattened to 24, 28, 32, and 36 months of age, respectively. From each group, ten healthy Yanbian cattle with similar body weights were randomly selected, the individual animal was used as the experimental unit, with pen as a random blocking factor. Slaughtered following standard operating procedures, with feed withheld for 12 h prior to slaughter. All animal experiments were conducted in accordance with the Guidelines for the Humane Treatment of Experimental Animals (issued by the Ministry of Science and Technology of the People’s Republic of China, Document No. [2006] 398). This study was reviewed and approved by the Laboratory Animal Welfare and Ethics Review Committee of Yanbian University (Approval No. YD20240827003). Immediately postmortem, the *longissimus dorsi* (LD) muscle at the sixth to eighth ribs of the left carcass was collected. After 24 h aging at 4 °C, meat quality indicators including color, water-holding capacity, and WBSF were measured immediately on fresh samples. For subsequent physical and chemical analyses, samples were rapidly frozen in liquid nitrogen and stored at −80 °C. Three 1 cm^2^ muscle tissue samples were obtained: the first was fixed in 4% paraformaldehyde solution for hematoxylin and eosin (H&E) staining to observe muscle fiber characteristics; the remaining two were fixed in 2.5% glutaraldehyde fixative at 4 °C for electron microscopy observation of sarcomeres and intramuscular connective tissue, respectively. Muscle tissue blocks measuring 0.5 × 0.5 × 0.2 cm were embedded in Optimal Cutting Temperature (OCT) compound within disposable base molds for ATPase staining of muscle fibers and Oil Red O staining to observe intramuscular fat distribution.

### 2.3. Slaughtering Performance Measurement

Fasting body weight (kg) was recorded on the day of slaughter, after 12 h of feed withdrawal, when each animal passed over a floor scale installed in the slaughter passageway. Following slaughter, commercial procedures were applied. After removal of the head, hooves, hide, and viscera, carcass weight (kg) was measured, and dressing percentage (%) was calculated. Backfat thickness (mm) was measured as the vertical thickness of subcutaneous fat on the left carcass. The outline of the LD muscle at the cross-section between the 6th and 8th ribs was traced onto sulfuric acid paper using a pencil, and the rib eye area (cm^2^) was calculated using the grid method. After precise dissection of the carcass, the total weight of boneless lean meat (kg) was recorded, and its percentage relative to live weight before slaughter (%) was calculated.

### 2.4. Meat Quality Traits Measurement

#### 2.4.1. Meat Color

Meat color was measured at 24 h postmortem after cutting to expose a fresh surface. A colorimeter (RID-20A, Shimadzu Corporation, Kyoto, Japan) equipped with an 8 mm aperture, illuminant D65, and 10° observer angle was calibrated against a standard white tile before each session. Three readings per sample were taken at different locations on the muscle surface at room temperature, and the average L*, a*, and b* values were recorded.

#### 2.4.2. pH

The pH values of muscle samples were measured at 45 min and 24 h after slaughter. Prior to the measurements, the pH meter (pH-100A, Shanghai Lichen Instrument Co., Ltd., Shanghai, China) was calibrated using standard buffer solutions of pH 4.0 and 7.0.

#### 2.4.3. Cooking Loss

Cooking loss was determined according to the method described by Hopkins et al. [16]. Meat samples were cut into uniform pieces weighing approximately 150.0 g and weighed (recorded as m1). The samples were placed in cooking bags and heated in a water bath at 80 °C until the core temperature reached 75 °C, and this temperature was maintained for an additional 20 min. After being removed and cooled to room temperature, the surface moisture was blotted dry with filter paper, and the samples were reweighed (recorded as m2). The cooking loss was calculated using the following formula:

(1) $$\mathrm{Cooking}\;\mathrm{loss}\;\left(\%\right)\;=\;(m1\;-m2)/m1\times 100$$

#### 2.4.4. Centrifugation Loss

Centrifugation loss was determined according to the method described by Li et al. [17]. Meat samples were cut parallel to the muscle fiber direction into strips (1 × 1 × 3 cm) weighing approximately 5.0 g, and weighed (recorded as m3). The samples were placed in centrifuge tubes and centrifuged at 2000× *g* for 15 min at 4 °C. After centrifugation, the exudate in the tubes was discarded. The residual fluid on the surface of the meat samples was blotted dry with filter paper, and the samples were reweighed (recorded as m4). The centrifugation loss was calculated using the following formula:

(2) $$\mathrm{Centrifugation}\;\mathrm{loss}\;\left(\%\right)\;=\;(m3\;-\;m4)/m3\times 100$$

#### 2.4.5. WBSF

The WBSF was measured on fresh samples after 24 h aging at 4 °C. LD muscle samples were cooked in 75 °C water baths to an internal temperature of 70 °C, then cooled to room temperature. Strips (1 × 1 × 3 cm) were cut parallel to the muscle fiber direction. A texture analyzer (TMS-PLUS, Food Technology Corporation, Sterling, VA, USA) equipped with a T-shaped blade (3.0 mm thickness, 90° cutting angle) sheared each strip perpendicular to the fiber direction. Testing parameters: crosshead speed 60 mm/min, trigger force 0.5 N. Three strips were measured per animal, and the average WBSF value was used for analysis.

### 2.5. Muscle Fiber Characteristics Measurement

#### 2.5.1. H&E Staining

After being fixed in a 4% formaldehyde solution for 48 h, the beef tissues were cut perpendicular to the muscle fiber direction into small blocks (1 × 1 × 0.5 cm) and rinsed with tap water overnight. The trimmed tissues were labeled, placed into embedding cassettes, and sequentially dehydrated through a graded ethanol series (70%, 80%, 90%, 95%, and 100%) for 1 h at each concentration. The samples were then cleared in xylene for 10 min, followed by immersion in soft and hard paraffin wax for 1 h each. After paraffin embedding, the blocks were sectioned at a thickness of 7 μm and subjected to H&E staining. The stained sections were observed and scanned using a microscope. For each animal, 20 muscle fibers were measured from a single H&E-stained section of the LD muscle. The muscle fiber cross-sectional area (S, μm^2^) was measured using ImageJ 1.8.0 software, and the muscle fiber diameter (L, μm) was calculated using the following equation:

(3) $$L\;=\;2\;\times \;\sqrt{\left(S/\pi \right)}$$

The muscle fiber density (D, num/mm^2^) was calculated based on the number of muscle fibers (N, num) within a specific unit area (S1, mm^2^) using the following equation:

(4) D = N/S1

#### 2.5.2. ATPase Histochemical Staining

Muscle fibers were typed using the acidic ATPase histochemical staining procedure according to the method described by Hwang et al. [18]. The cryo-embedded tissues were sectioned using a cryostat at a thickness of 8 μm. The sections were incubated in an acidic ATPase working solution (49 mL of 0.2 mol/L sodium acetate and 45 mL of 0.2 mol/L glacial acetic acid, pH 4.63) at 37 °C for 10 min. Subsequently, the sections were rinsed three times with an ATPase incubation medium (prepared by mixing 5 mL of the aforementioned working solution, 0.6 g of disodium ATP, and 15 mL of distilled water, adjusted to pH 9.5), and then incubated in this medium at 37 °C for 2 h. After incubation, the sections were sequentially treated with a 1% calcium chloride solution for 6 min and a 2% cobalt chloride solution for 5 min, followed by rinsing three times with distilled water. The sections were then developed in an ammonium sulfide solution for 1 min. After being rinsed three times with tap water, the sections were dehydrated with ethanol, cleared in xylene, mounted with neutral balsam, and scanned for observation under a microscope. Following this acidic pre-incubation, type I fibers appeared dark brown, while type II fibers appeared light gray or colorless. The percentage of type I and type II fibers was calculated for each animal by ImageJ 1.8.0 software. The animal was used as the experimental unit (n = 10 per fattening stage).

#### 2.5.3. Transmission Electron Microscopy (TEM) Observation

The samples fixed in 2.5% glutaraldehyde were immersed in a phosphate buffer for 2 h and subsequently dehydrated through a graded ethanol series. The samples were placed into embedding molds, embedded in resin, and polymerized in an oven at 70 °C for 48 h. After the resin was fully cured, the embedded blocks were extracted, trimmed, and cut into approximately 70 nm thick ultrathin sections. The sections were stained with uranyl acetate and lead citrate for 10 min. After drying, the sections were observed, and images were captured using a TEM (RTTLIT E20, Zansun Optoelectronics Technology Co., Ltd., Suzhou, China).

### 2.6. Intramuscular Fat (IMF) Content

#### 2.6.1. Determination of Fat Content

A 2.0 g minced meat sample was accurately weighed and mixed with 25 mL of a chloroform-methanol solution (2:1, *v*/*v*), followed by homogenization for 1 min. An additional 15 mL of the same solvent mixture was added. The mixture was thoroughly mixed and allowed to stand for 1 h. After filtration, 8.8 mL of an aqueous solution containing 7.3 g/L NaCl and 0.5 g/L CaCl_2_ was added to the filtrate. The mixture was then centrifuged at 3000× *g* for 15 min. The supernatant was discarded, and the lower layer was collected, concentrated using a rotary evaporator, and dried to a constant weight. The fat content was calculated based on the weight difference in the flask before and after evaporation.

#### 2.6.2. Oil Red O Staining

Beef samples embedded in OCT were sectioned at a thickness of 7 μm using a cryostat. The sections were rinsed in 60% isopropanol for 30 s. An Oil Red O working solution was prepared by mixing 6 mL of Oil Red O stock solution with 4 mL of distilled water. After standing for 10 min, the sections were stained with this solution for 8 min. Following staining, excess dye was washed away with 60% isopropanol, and the sections were rinsed with distilled water. The moisture surrounding the sections was blotted dry with filter paper, and the slides were mounted using a specialized mounting medium.

### 2.7. Intramuscular Connective Tissue Characteristics

#### 2.7.1. Scanning Electron Microscope (SEM) Observation

The structural morphology of the beef intramuscular connective tissue was observed using an SEM according to the method described by Roy and Bruce [19]. The samples were removed from the 2.5% glutaraldehyde fixative and rinsed three times in a phosphate buffer. Subsequently, the samples were immersed in a 2 mol/L NaOH solution at 4 °C for 7 d. During this period, the NaOH solution was replaced twice daily until the tissues became transparent. After being soaked in deionized water for 2 h, the samples were immersed in a 1% tannic acid solution for 4 h, followed by thorough rinsing with deionized water. The samples were then re-fixed in 2.5% glutaraldehyde for 2 h. Following fixation, the samples were dehydrated through a graded ethanol series, substituted with tert-butanol, and dried using a vacuum freeze dryer. The dried samples were sputter-coated with gold, and images of the intramuscular connective tissue structure were captured using an SEM (SU 8010, Hitachi, Tokyo, Japan).

#### 2.7.2. Determination of Total Collagen Content and Thermal Solubility

The contents of total collagen and heat-soluble collagen were calculated based on the hydroxyproline content. The determination of hydroxyproline content was outsourced to Beijing BiotechPack Scientific Co., Ltd. (Beijing, China). Accurately weigh 10.0 mg of the beef sample, add 800 μL of ultrapure water for grinding, and centrifuge to obtain the supernatant. Add 1 mL of ultrapure water to make up the volume of the supernatant to 1 mL, then add 1 mL of 6 mol/L hydrochloric acid. Acid hydrolyzes at 110 °C for 24 h. After hydrolysis, take 1 mL of the liquid, evaporate it, add 1 mL of ultrapure water, and shake for 1 h. Centrifuge at 4 °C, 12,000× *g* for 10 min to collect the supernatant. Take 20 μL of the supernatant and add it to a 1.5 mL centrifuge tube. Add 5 μL of internal standard solution and 40 μL of isopropanol. Vortex for 2 min. Centrifuge at 4 °C, 12,000× *g* for 10 min, and then transfer 10 μL of the supernatant to a new centrifuge tube. Add 70 μL of borate buffer salt and 20 μL of AccQ Tag derivatization reagent and vortex for 10 s. After standing, heat at 55 °C for 10 min to complete the derivatization reaction. Add 400 μL of water for dilution. Take 100 μL of the diluted solution for analysis.

The chromatographic separation was carried out using the Waters ACQUITY UPLC I CLASS system. The chromatographic column was Waters UPLC HSS T3 (1.8 μm, 2.1 mm × 150 mm). The mobile phase A was a water solution containing 0.1% formic acid, and B was acetonitrile. The flow rate was 0.5 mL/min, the injection volume was 5.0 μL, and the column temperature was 50 °C. The mass spectrometry detection was carried out using the XEVO TQ-S Micro tandem quadrupole mass spectrometry system (Waters Technology Co., Ltd., Shanghai, China). The positive ion mode was used, with a voltage of 1.5 kV, a cone voltage of 20 V, a desolvation temperature of 600 °C, gas flow rate of 1000 L/h, and a cone gas flow rate of 10 L/h.

Data processing was carried out using the MassLynx 4.1 quantitative software. The target peak was identified by retention time, and the initial quantitative value of hydroxyproline was obtained using the standard curve method. The content was calculated as follows:

(5) Hydroxyproline content = (V × M1 × n)/(1000 × M2)

(6) Collagen content = Hydroxyproline content × 7.46

Hydroxyproline content: mg/g meat; V: initial quantitative value; M1: molar mass of hydroxyproline; n: dilution factor; M2: sample mass; Collagen content: mg/g meat tissue.

Heat-soluble collagen was extracted from the beef samples using a Ringer’s solution dissolution method, according to the procedure described by Kim et al. [20]. The resulting supernatant was analyzed following the hydroxyproline quantification protocol. The heat-soluble collagen content was then calculated based on the measured hydroxyproline content, which was subsequently used to evaluate the thermal solubility of the collagen.

(7) Collagen solubility (%)=(Heat-soluble collagen/Total collagen) × 100

#### 2.7.3. Determination of Hydroxylysine Pyridinoline (HP) Cross-Link Content

The content of HP cross-links was determined using a commercial assay kit (Shanghai Huabang Biotechnology Co., Ltd., Shanghai, China) according to the manufacturer’s instructions. Beef samples were mixed with 10 volumes of ice-cold PBS and thoroughly homogenized in an ice bath. The homogenate was then centrifuged at 3000× *g* for 20 min at 4 °C, and the supernatant was collected for subsequent analysis. To establish the standard curve, the standards provided in the kit were prepared into a dilution series with concentrations of 800, 400, 200, 100, 50, and 0 pg/mL. The absorbance was measured at a wavelength of 450 nm, and a standard curve was generated. The PBS-extractable HP cross-links content in the supernatant was quantified strictly following the procedures detailed in the kit protocol. Values are expressed as nanograms of HP per gram of meat tissue.

### 2.8. Statistical Analysis

Data collation and preprocessing were performed using Microsoft Excel 2021. Subsequent statistical analyses were conducted using SPSS 27.0 software. The significance of differences between groups was analyzed using a one-way analysis of variance (ANOVA), followed by Tukey’s honestly significant difference (HSD) test for multiple comparisons. The data are presented in the form of mean ± standard deviation (Mean ± SD), and the level of significance was set at *p* < 0.05. The individual animal was the experimental unit (n = 10 per fattening stage). For image-based traits, multiple measurements per animal were averaged, and the animal-level average was used in one-way ANOVA. This approach avoids pseudo-replication and is appropriate for balanced designs with animal as the experimental unit. Normality was assessed using the Shapiro–Wilk test (*p* > 0.05). Homogeneity of variances was assessed using Levene’s test (*p* > 0.05). Different lowercase letters in the tables indicate significant differences between groups. All graphs were generated using GraphPad Prism 10.0 software, with statistical significance levels denoted by asterisks: * *p* < 0.05, ** *p* < 0.01, and *** *p* < 0.001.

## 3. Results

### 3.1. Slaughtering Performance Analysis

Slaughter performance indicators of Yanbian cattle at different fattening periods were determined, as shown in Table 2. A significant overall effect of fattening period was detected for pre-slaughter live weight (*p* < 0.05). Pairwise comparisons showed that pre-slaughter live weight increased progressively with age, with all inter-group differences being significant (*p* < 0.05 for all comparisons between 24, 28, 32, and 36 months), indicating that the entire fattening stage is a period of rapid body weight accumulation. Similarly, a significant overall effect of fattening period was detected for both carcass weight, net meat weight, and backfat thickness (*p* < 0.05 for all inter-group comparisons). In the 36-month-old group, the carcass weight reached 424.97 ± 12.82 kg, and the average net meat weight was 319.94 ± 11.11 kg. The dressing percentage increased from 56.06 ± 1.39% at 24 months of age to 60.09 ± 1.43% at 32 months of age (*p* < 0.05), with no significant difference between the 32- and 36-month groups (*p* > 0.05). A significant overall effect of fattening period was also detected for net meat percentage (*p* < 0.05). Pairwise comparisons revealed that net meat percentage was significantly lower at 24 months than at 28, 32, and 36 months (*p* < 0.05 for all). The highest net meat percentage was observed in the 32-month-old group (46.96 ± 0.53%), which was significantly higher than both the 28- and 36-month groups (*p* < 0.05). No significant difference was detected between 28 and 36 months (*p* > 0.05). Pairwise comparisons showed that ribeye area was significantly lower in the 24- and 28-month groups compared to the 32- and 36-month groups (*p* < 0.05 for all comparisons).

### 3.2. Meat Quality Traits Analysis

The results of the meat quality of the LD muscle from Yanbian cattle at different fattening periods are presented in Table 3. Among the meat color parameters, no significant effect of fattening period on b* values was detected (*p* > 0.05). Significant overall effects of fattening period were detected for both L* and a* values (*p* < 0.05). Pairwise comparisons revealed that the L* value was significantly higher in the 24-month group than in the 28-, 32-, and 36-month groups (*p* < 0.05 for all comparisons). Conversely, the a* value was significantly lower in the 24-month group compared to the other three groups (*p* < 0.05 for all comparisons). The pH_45 min_ value, which reflects the initial rate of postmortem glycolysis, showed no significant differences among the groups (*p* > 0.05). The normal range of pH 24 h (5.61–5.67) across all groups confirms that the observed differences in water-holding capacity and WBSF are attributable to fattening stage rather than to aberrant postmortem glycolysis. Throughout the entire fattening period, the cooking loss exhibited a trend of initially increasing and then decreasing. Among the groups, the LD muscle from the 28-month-old Yanbian cattle recorded the highest value. For centrifugation loss, a significant overall effect of fattening period was detected (*p* < 0.05). Pairwise comparisons showed that centrifugation loss was significantly higher in the 24- and 28-month groups than in the 32- and 36-month groups (*p* < 0.05 for all comparisons), indicating that the beef from the 32- and 36-month-old cattle possessed the best mechanical water-holding capacity. The WBSF value for the 24-month-old group was 68.42 ± 9.17 N, which was significantly lower than those of the 32- and 36-month-old groups (*p* < 0.05). The data indicated that starting from 32 months of age, the shear force increased significantly (*p* < 0.05) and remained at a relatively high level.

### 3.3. Muscle Fiber Analysis

#### 3.3.1. Muscle Fiber Characteristics

**Figure 1:**
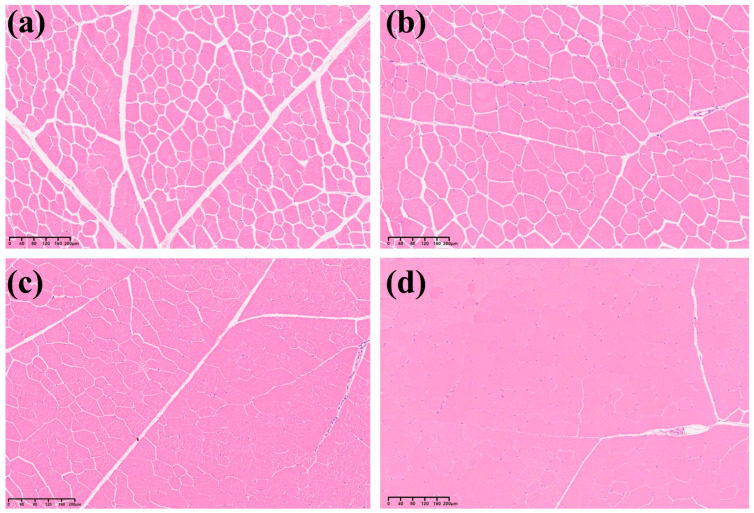
Muscle fibers H&E staining of Yanbian cattle during the fattening periods. (Scale = 200 μm). (**a**–**d**) Respectively represent beef samples at 24, 28, 32 and 36 months of age.

**Figure 2:**
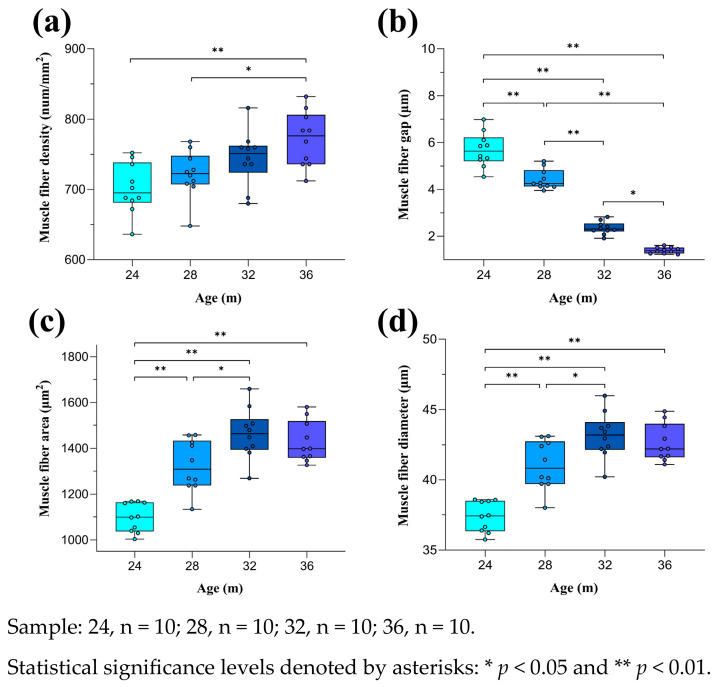
Muscle fiber characteristics changes in Yanbian cattle during the fattening periods. (**a**) Muscle fiber density, (**b**) Muscle fiber gap, (**c**) Muscle fiber area, and (**d**) Muscle fiber diameter.

To elucidate the underlying mechanisms driving the differences in meat quality of Yanbian cattle across different fattening periods, the histological morphology of muscle fibers in the LD muscle was observed using H&E staining (Figure 1). Subsequently, parameters including muscle fiber density, cross-sectional area, and intermuscular fiber gap were measured, with the results presented in Figure 2. As shown in Figure 1, the muscle fibers of 24-month-old Yanbian cattle were loosely arranged and slender, with noticeable gaps visible between the muscle bundles. As the fattening period progressed, the muscle fiber diameter began to increase, the arrangement became more compact, and the inter-bundle spaces decreased significantly.

#### 3.3.2. Muscle Fiber Type

**Figure 3:**
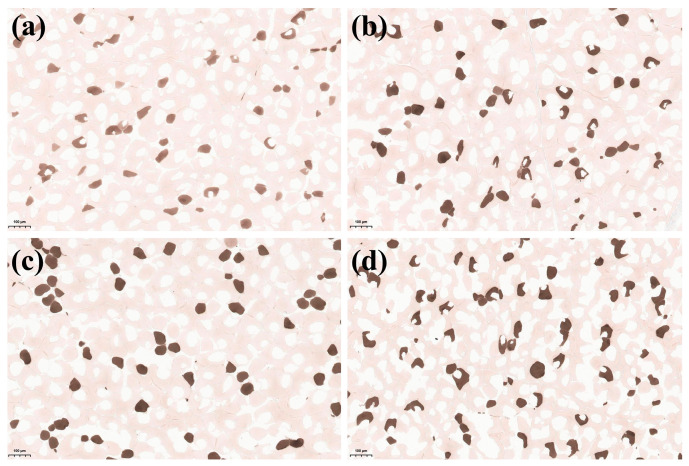
ATPase histological staining in muscle fiber types of Yanbian cattle during the fattening periods. (Scale = 100 μm). (**a**–**d**) Respectively represent beef samples at 24, 28, 32 and 36 months of age.

To investigate the muscle fiber type composition of the LD muscle in Yanbian cattle across different fattening periods, acid-incubated ATPase enzyme histochemical staining was performed. Type I muscle fibers, exhibiting higher acid resistance of ATPase, were stained dark brown, while type II muscle fibers appeared light gray or colorless. As shown in Figure 3, the muscle fiber type composition displayed a mixed structure with interspersed distribution of fast- and slow-twitch fibers, with type II muscle fibers being more abundant. The 24-month-old group predominantly exhibited type II muscle fibers, with type I fibers sparsely distributed among them. The data in Table 4 shows that as the fattening period progressed, the number of type I muscle fibers increased from 19.31% at 24 months to 36.20% at 36 months, indicating a transition of the muscle toward slow-contraction characteristics.

#### 3.3.3. Myofibrillar Ultrastructure

**Figure 4:**
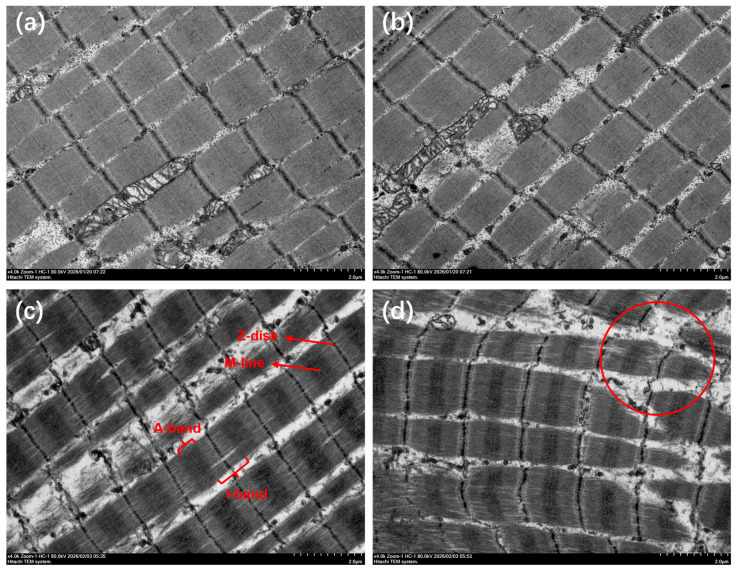
TEM observation of muscle sarcomere of Yanbian cattle during the fattening periods. (Scale = 2 μm). (**a**–**d**) Respectively represent beef samples at 24, 28, 32 and 36 months of age.

In this study, TEM was employed to observe the sarcomere morphology and ultrastructure of beef. As shown in Figure 4 and Table 5, sarcomeres exhibited generally consistent lengths in the LD muscle of Yanbian cattle across different fattening periods (*p* > 0.05). Although sarcomere length remained stable, certain changes in myofibrillar structure were observed. Most notably, in beef samples from the later fattening period, predominantly at 36 months of age, adipocytes appeared in the spaces between myofibrils. These adipocytes presented as vacuole-like structures under electron microscopy, embedded between myofibril bundles, causing disruptions between adjacent Z-disks (As shown by the circle in Figure 4d).

### 3.4. Intramuscular Fat Content Analysis

**Figure 5:**
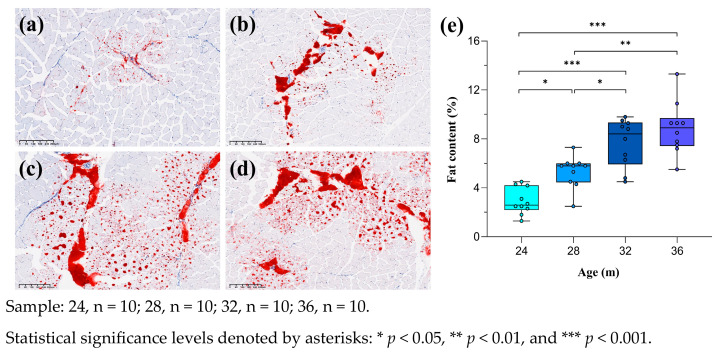
Intramuscular fat oil red O staining (**a**–**d**) and fat content (**e**) of Yanbian cattle during the fattening periods. (Scale = 300 μm).

To investigate the dynamic process of intramuscular fat formation in Yanbian cattle beef during different fattening periods, Oil Red O staining was performed to visualize adipocytes, and fat content was measured. As shown in Figure 5e, the effect of fattening period on intramuscular fat content was detected (*p* < 0.001). Specifically, fat content was higher at 32 months than at 24 months (*p* < 0.001) and 28 months (*p* < 0.05). Fat content at 36 months was higher than at 24 months (*p* < 0.001) and 28 months (*p* < 0.01). There was no statistically significant difference between the 36- and 32-month-old groups (*p* > 0.05). As the fattening period progressed, adipocytes transitioned from sporadic distribution to aggregation and connection, gradually filling the inter-bundle spaces and ultimately forming a marbling structure. As shown in Figure 5, 28 months of age represented a critical turning point for intramuscular fat deposition, with a marked increase in the number of adipocytes exhibiting distinct cluster-like aggregation. During the mid-to-late fattening period, fat droplets in the LD samples from 32 and 36 months of age were observed in the perimysial and endomysial spaces. This resulted in a more homogeneous admixture of adipose and muscle tissues, where fat is no longer confined to the exterior of muscle bundles but instead infiltrates between individual muscle fibers.

### 3.5. Intramuscular Connective Tissue Analysis

#### 3.5.1. Connective Tissue Characteristics

**Figure 6:**
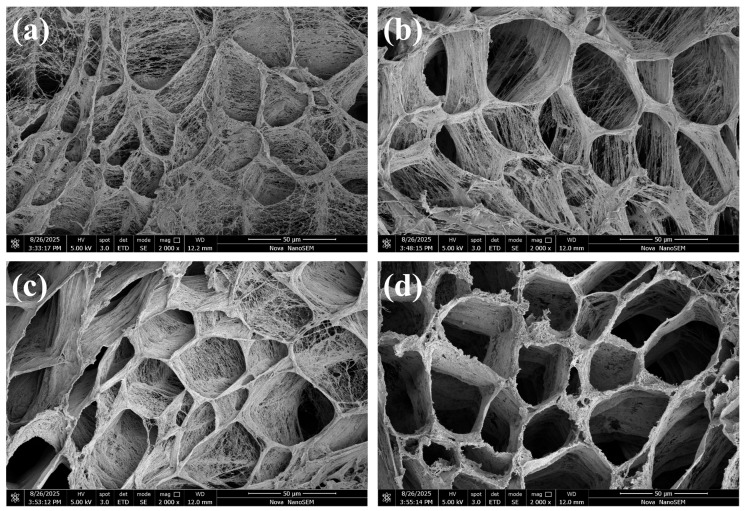
SEM of muscle connective tissue of Yanbian cattle during the fattening periods. (Scale = 50 μm). (**a**–**d**) Respectively represent beef samples at 24, 28, 32 and 36 months of age.

In this study, SEM was employed to observe the ultrastructure of endomysial collagen fibers in the LD muscle of Yanbian cattle at different fattening periods, with results shown in Figure 6. At the early fattening stage (24 months of age), the endomysial collagen fibers exhibited a slender, filamentous distribution, with irregular and random orientation, forming a loosely organized three-dimensional network. Fiber density was low, inter-fiber spaces were relatively large, and the compactness of the network structure was insufficient. This ultrastructure corresponded to a lower background toughness, consistent with the better tenderness observed in 24-month-old cattle beef. As the fattening period extended, the collagen fibers began to thicken, fiber density markedly increased, inter-fiber spaces decreased significantly compared to 24 months, and the network structure became more compact. In the 32-month-old group, some collagen fibers exhibited aggregation, showing a transition from a filamentous to a sheet-like morphology.

#### 3.5.2. Collagen Content and Its Solubility

**Figure 7:**
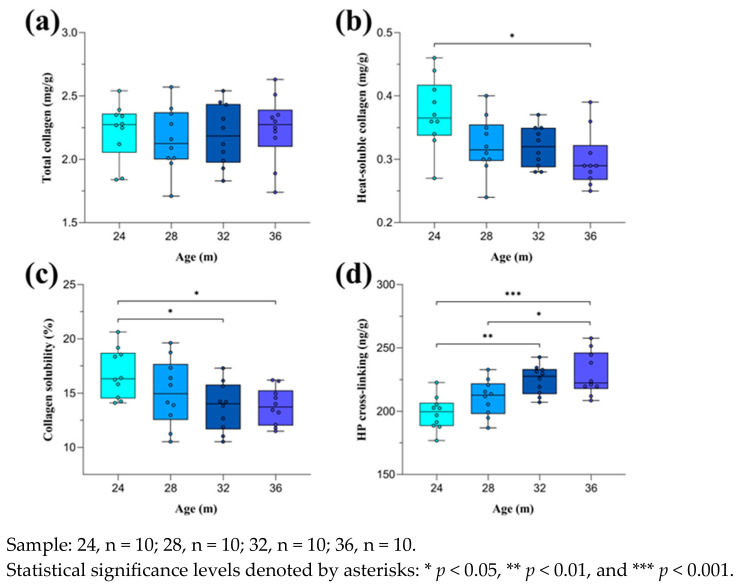
Changes in total collagen content (**a**), heat-soluble collagen content (**b**), collagen solubility (**c**) and HP cross-linking content (**d**) of Yanbian cattle during the fattening periods.

To validate the ultrastructural changes in connective tissue, the total collagen content and heat-soluble collagen content were measured in LD muscle samples of Yanbian cattle at different fattening periods (Figure 7a–c). The results showed no significant difference in total collagen content among the groups during the fattening period (*p* > 0.05). The effect of fattening period on collagen solubility was detected. Specifically, heat-soluble collagen content was higher at 24 months than at 36 months (*p* < 0.05). Collagen solubility was higher at 24 months than at 32 months (*p* < 0.05) and 36 months (*p* < 0.05), with collagen heat solubility decreasing by 3.07% from 24 to 36 months of age.

#### 3.5.3. HP Cross-Linking Content

An effect of fattening period on HP cross-link content was detected (*p* < 0.001). Specifically, the HP cross-link content at 24 months of age had the lowest value (195.54 ± 12.47 ng/g) throughout the fattening period, indicating a relatively low degree of mature cross-linking of collagen fibers and weaker thermal stability of the collagen network at this stage. The HP cross-link contents in the 28- and 32-month-old groups were 210.57 ± 13.50 ng/g and 224.78 ± 10.86 ng/g, respectively, with no significant difference between them (*p* > 0.05). This gradual increase marked the initiation of collagen fiber cross-linking construction and the beginning of enhanced collagen maturity. By the end of the fattening period, the HP cross-link content reached its peak at 229.62 ± 16.19 ng/g. Compared with the 24-month-old group, this significant increase signified the maturation and strengthening of the collagen fiber cross-linking network.

## 4. Discussion

Slaughter performance is an essential criterion for evaluating the meat production capacity of beef cattle, directly reflecting the fattening efficacy [21]. Slaughter performance results indicate that prolonging the fattening time within the experimental period has a significant positive effect on enhancing the meat yield of beef cattle. Although muscle tissue development continued between 32 and 36 months of age, its growth rate had reached a plateau. Meanwhile, the results showed that backfat thickness also continuously increased with the extension of the fattening period. This dynamic fully confirms that the body’s capacity for fat deposition is enhanced as the fattening cycle is prolonged. This finding is consistent with the results of Moloney et al. [22], who compared the carcass and meat quality characteristics of cattle raised on pasture or indoors to 30 months of age. Their results showed that although 30-month-old cattle had greater body weight, the growth rate of muscle fibers had already significantly slowed, and the meat quality was characterized primarily by increased fat deposition rather than a significant improvement in lean meat percentage.

The lightness or darkness of meat color primarily depends on the content and chemical state of myoglobin within the muscle. Myoglobin is an oxygen-storing pigment protein in the muscle, and its concentration increases as the animal ages [23]. Therefore, the darker meat color and higher redness observed in the 36-month-old Yanbian cattle reflects an increased accumulation of myoglobin. The findings of Jerez-Timaure et al. [24] support the observation in this study that there was no significant difference in pH among different fattening periods of Yanbian cattle. In their study on grass-fed yellow cattle, no statistically significant differences in postmortem pH values were found among different age groups.

In the meat quality evaluation system, water-holding capacity (WHC) is defined as the ability of muscle tissue to resist moisture loss and retain water under the influence of external conditions such as heating, the application of pressure, freezing, and thawing [25]. Cooking loss reflects the degree of moisture loss from the muscle during simulated thermal processing [26]. The progressive decline in centrifugation loss with fattening age may be multifactorial. Ultimate pH_24 h_ did not differ among groups (*p* > 0.05), ruling out pH-mediated effects. The gradual accumulation of intramuscular fat may have physically limited the space available for free water, potentially reducing moisture expulsion under centrifugal force [27]. Additionally, increased collagen cross-linking at later fattening stages may have provided greater structural integrity, potentially minimizing centrifugal moisture loss [28]. Other factors, including protein denaturation, postmortem aging were identical across groups. Thus, IMF accumulation and collagen maturation likely played contributory roles in the observed improvement in water-holding capacity.

Similar to the results of this study, Jeong et al. [29] compared the meat quality characteristics of Hanwoo cattle at 26 vs. 31 months fattening stages. Their results showed that with increasing age, the WBSF values exhibited significant fluctuations and tended to stabilize at a relatively high level. This finding suggests that as cattle age beyond approximately 30 months, the connective tissue within the muscle likely increases, leading to elevated meat toughness.

The histological characteristics of muscle constitute the microstructural basis determining meat quality traits such as tenderness and water-holding capacity. As the fattening period progressed, the muscle fiber diameter began to increase, the arrangement became more compact, and the inter-bundle spaces decreased significantly. This trend was concurrent with changes in macroscopic meat yield indicators such as ribeye area and lean meat percentage, indicating that muscle fiber hypertrophy serves as the microscopic basis for increased muscle production [30]. The observed decrease in muscle fiber area may be associated with increased IMF deposition [31]. Speculated that when large amounts of IMF accumulate, adipocytes occupy a portion of the spatial volume, which may result in a relative reduction in the area occupied by muscle fibers in the microscopic field.

The composition of muscle fiber types serves as the histological basis for determining the metabolic characteristics, contractile properties, and meat quality traits of skeletal muscle. Typically, as animals age, skeletal muscle tends to transition toward type II fibers, which exhibit faster contraction speeds and greater glycolytic capacity [32]. However, the results of the present study showed a continuous increase in type I muscle fibers. One possible explanation is that adipokines secreted by adipocytes may influence the metabolic properties of surrounding muscle fibers via paracrine pathways, potentially inducing conversion toward the oxidative type I phenotype [33]. Another hypothesis is that as Yanbian cattle continue to gain body weight with increasing fattening time, the LD muscle, as a core postural muscle, may be required to bear greater mechanical loads, which could promote the transformation of muscle fibers toward slow-twitch or oxidative fast-twitch types [34]. Further studies using molecular and functional approaches are needed to test these speculative mechanisms. Muscle fiber type is closely associated with beef tenderness and texture. Studies have shown that muscles dominated by fast-twitch type II fibers are more prone to proteolysis during the early postmortem period compared to those dominated by slow-twitch type I fibers [35]. Additionally, type II fibers were reported to significantly influence the rate and extent of postmortem pH decline, making them a key factor determining ultimate beef tenderness due to their abundant glycogen reserves [6]. Therefore, the increase in the number of type I muscle fibers during the fattening period may contribute to the reduction in beef tenderness, although the underlying mechanisms remain to be elucidated.

Sarcomere length reflects the contractile state of muscle tissue or the degree of myofilament sliding during postmortem aging. Longer sarcomeres indicate a more relaxed myofibrillar network and generally predict more tender meat [36]. However, results indicate that variation in sarcomere length was not the primary factor contributing to differences in tenderness during the fattening period. During the fattening period, the ultrastructure of the LD muscle in Yanbian cattle exhibited overall sarcomere stability with localized fat deposition. These processes lead to ultrastructural alterations in beef at the end of the fattening period, providing a microscopic basis for explaining beef texture characteristics.

Marbling is indicative of intramuscular fat content and its distribution pattern within the muscle. Although there was no statistically significant difference between the 36- and 32-month-old groups, the overall trend continued to increase, indicating that the fat deposition process persisted throughout the entire fattening period. The result is also consistent with the concept of a marbling plateau reported by Kern et al. [37]. These authors noted that marbling content will reach a plateau without the recruitment of additional adipocytes, suggesting that the plateau phase between 32 and 36 months in Yanbian cattle may indicate a temporary balance between adipocyte hyperplasia and hypertrophy.

As a major component of muscle, intramuscular connective tissue is primarily composed of collagen fibers. Its content, degree of cross-linking, and spatial arrangement constitute the “background toughness” of beef [38]. By the end of the fattening period, the endomysial collagen fibers reached their most mature state. The original filamentous morphology had essentially disappeared, and the fibers were completely fused, replaced by a dense sheet-like structure [39]. This ultrastructure corresponded to a significantly increased background toughness, consistent with the marked elevation in shear force values observed at 36 months of age. Consistent with the findings of Nishimura et al. [38] on beef, with increasing age, the collagen fibrils associate more closely with each other, the collagen fibers within the perimysium increase in thickness. These changes are closely associated with an increase in the mechanical strength of the intramuscular connective tissue and contribute to the toughening of beef during this period.

The results showed no significant difference in total collagen content; this indicates that the morphological changes in endomysial collagen fibers observed by scanning electron microscopy may not be attributable to increased collagen synthesis, but rather to structural remodeling and enhanced cross-linking of collagen fibers. Heat-soluble collagen refers to the fraction of collagen that can be solubilized from muscle tissue upon heating, including newly synthesized collagen that has not formed stable covalent cross-links and collagen fibers with a low degree of cross-linking. Its content directly reflects the maturity and thermal stability of the collagen network [40,41]. Heat-soluble collagen content, and the collagen solubility reflected thereby, exhibited a significant decreasing trend as the fattening period extended. This suggests that the fraction of collagen fibers susceptible to heat-induced denaturation and degradation gradually decreased during muscle development.

HP cross-linking is the primary form of mature collagen cross-linking in collagen fibers. As a trifunctional cross-link, HP forms covalent bonds connecting adjacent collagen molecules, thereby conferring mechanical strength and thermal stability to collagen fibers [42]. The HP cross-link content at 24 months of age had the lowest value, indicating a relatively low degree of mature cross-linking of collagen fibers and weaker thermal stability of the collagen network at this stage. This finding is highly consistent with the higher collagen solubility and lower background toughness observed. The increase in HP cross-link content directly corresponded to the morphological transformation of collagen fibers observed by scanning electron microscopy. Studies have shown that beef cattle breeds with fast growth rates and early slaughter ages typically exhibit lower degrees of collagen cross-linking and more tender meat [40], whereas indigenous breeds with slow growth rates and late slaughter ages display higher degrees of collagen cross-linking and meat with greater chewiness. As a renowned indigenous Chinese breed, the HP cross-link levels and their changing patterns in Yanbian cattle reflect the unique mechanisms underlying its meat quality formation.

Although IMF increased with fattening, WBSF also increased at 32 and 36 months. This apparent paradox is explained by the overriding effect of collagen maturation (decreased solubility, increased HP cross-linking) on WBSF. The magnitude of change in collagen properties likely has a greater impact on WBSF than the modest increase in IMF. Furthermore, perimysial adipocytes may not effectively disrupt the highly cross-linked endomysial collagen network during shearing. Therefore, in Yanbian cattle, collagen-mediated toughening dominates at later fattening stages, and 32 months represents a trade-off between marbling/yield and the onset of increased toughness, rather than an improvement in tenderness. This finding provides key evidence for understanding the underlying mechanisms of tenderness deterioration in Yanbian beef during the late fattening period and holds important reference value for determining the optimal slaughter age.

## 5. Conclusions

This study systematically evaluated slaughter performance, meat quality, and histochemical characteristics of Yanbian cattle at different fattening periods. The results demonstrate that 32 months of age represents the optimal fattening endpoint, as beyond this age the proportion of inedible fat in additional weight gain likely exceeds that of muscle tissue. Regarding meat quality, the 32- and 36-month-old groups exhibited the best water-holding capacity. Type I muscle fibers increased significantly with age, indicating a shift toward oxidative metabolism. The endomysial collagen network at 36 months had transformed into a dense sheet-like configuration, increasing background toughness. Heat-soluble collagen and HP cross-link levels at 32 months indicated a mature but not excessively stabilized collagen network. In conclusion, fattening to 32 months achieves a compromise among carcass yield, intramuscular fat deposition, water-holding capacity, collagen maturity, and the onset of increased WBSF. Therefore, 32 months is recommended as the optimal fattening endpoint for Yanbian cattle to maximize meat quality.

## Figures and Tables

**Table 1:** Dietary composition and nutritional levels (Dry matter basis).

Ingredient Composition	Content %	Nutritional Levels	Content %
Maize straw	25.0	DM	85.72
Corn meal	48.0	CP	14.51
Soybean meal	16.0	EE	4.27
Wheat bran	5.5	Ash	5.09
NaHCO_3_	0.6	NDF	28.56
NaCl	0.5	ADF	16.76
Compound premix ^1^	4.3	Ca	0.50
Total	100	P	0.32
		Neg ^2^ (Mcal/kg)	1.32

^1^ The compound premix provided the following per kilogram: iron, 1000–2000 mg; copper, 200–500 mg; zinc, 600–1200 mg; manganese, 800–2000 mg; iodine, 1050 mg; cobalt, 2–10 mg; selenium, 2–10 mg; vitamin A, 20,000 IU; vitamin D, 80,000 IU; vitamin E, ≥200 mg; vitamin K, ≥10 mg; vitamin H, ≥2 mg; ethoxyquin, ≤100 mg; lactic acid bacteria, 1 × 10^11^ CFU; live yeast, 6 × 10^10^ CFU; and Bacillus, 5 × 10^10^ CFU. ^2^ Net energy for gain was estimated from the analyzed values of the dietary components. Abbreviations: DM, dry matter; CP, crude protein; EE, ether extract; ADF, acid detergent fiber; NDF, neutral detergent fiber; Ca, calcium; P, phosphorus.

**Table 2:** The influence of fattening period on the slaughtering performance of Yanbian cattle.

Items	Month of Age (m)			
	24	28	32	36
Body weight (kg)	499.82 ± 15.53 ^d^	558.80 ± 11.01 ^c^	649.22 ± 21.02 ^b^	710.22 ± 15.67 ^a^
Carcass weight (kg)	280.20 ± 10.90 ^d^	321.18 ± 9.77 ^c^	390.09 ± 15.19 ^b^	424.97 ± 12.82 ^a^
Dressing percentage (%)	56.06 ± 1.39 ^c^	57.47 ± 1.00 ^b^	60.09 ± 1.43 ^a^	59.83 ± 1.20 ^a^
Net meat (kg)	215.74 ± 11.01 ^d^	248.91 ± 8.98 ^c^	304.86 ± 9.21 ^b^	319.94 ± 11.11 ^a^
Net meat percentage (%)	43.16 ± 1.61 ^c^	44.53 ± 0.94 ^b^	46.96 ± 0.53 ^a^	45.04 ± 0.78 ^b^
Rib eye area (cm^2^)	92.45 ± 13.59 ^b^	97.54 ± 10.20 ^b^	108.68 ± 8.10 ^a^	116.68 ± 7.76 ^a^
Backfat thickness (mm)	9.29 ± 1.00 ^d^	10.54 ± 1.30 ^c^	11.60 ± 1.17 ^b^	13.07 ± 0.94 ^a^

Data are presented as mean ± SD. Sample: 24, n = 10; 28, n = 10; 32, n = 10; 36, n = 10. Different lowercase letters in the tables indicate significant differences between groups (*p* < 0.05).

**Table 3:** The influence of fattening period on the meat quality traits of Yanbian cattle.

Items	Month of Age (m)			
	24	28	32	36
L*	36.20 ± 2.04 ^a^	30.33 ± 1.64 ^b^	30.67 ± 2.06 ^b^	29.09 ± 2.06 ^b^
a*	16.04 ± 3.26 ^b^	20.06 ± 1.84 ^a^	19.54 ± 1.67 ^a^	20.59 ± 2.53 ^a^
b*	9.57 ± 0.68	9.22 ± 0.54	9.44 ± 1.11	9.26 ± 0.90
pH_45 min_	6.77 ± 0.10	6.74 ± 0.11	6.75 ± 0.16	6.74 ± 0.15
pH_24 h_	5.67 ± 0.17	5.65 ± 0.13	5.61 ± 0.11	5.63 ± 0.18
Cooking loss (%)	32.25 ± 2.69 ^ab^	33.63 ± 3.17 ^a^	31.12 ± 1.74 ^ab^	29.81 ± 2.53 ^b^
Centrifugation loss (%)	1.80 ± 0.13 ^a^	1.65 ± 0.06 ^a^	1.45 ± 0.10 ^b^	1.43 ± 0.07 ^b^
WBSF (N)	68.42 ± 9.17 ^b^	72.83 ± 13.87 ^b^	85.54 ± 14.31 ^a^	85.75 ± 10.06 ^a^

Data are presented as mean ± SD. Sample: 24, n = 10; 28, n = 10; 32, n = 10; 36, n = 10. Different lowercase letters in the tables indicate significant differences between groups (*p* < 0.05). L*: Lightness; a*: Green–Red Axis; b*: Blue–Yellow Axis; WBSF: Warner–Bratzler shear force.

**Table 4:** The percentage of type I and type II fibers of Yanbian cattle during fattening periods.

Items	Month of Age (m)			
	24	28	32	36
Type I fibers (%)	19.31 ± 3.27 ^c^	24.87 ± 4.28 ^b^	26.12 ± 3.60 ^b^	36.20 ± 2.71 ^a^
Type II fibers (%)	78.19 ± 6.34 ^a^	75.21 ± 5.32 ^ab^	70.54 ± 8.11 ^b^	58.62 ± 9.07 ^c^

Data are presented as mean ± SD. Sample: 24, n = 10; 28, n = 10; 32, n = 10; 36, n = 10. Different lowercase letters in the tables indicate significant differences between groups (*p* < 0.05).

**Table 5:** The sarcomere length of Yanbian cattle during fattening periods.

Items	Month of Age (m)			
	24	28	32	36
Sarcomere length (μm)	1.82 ± 0.23	1.79 ± 0.21	1.73 ± 0.32	1.80 ± 0.17

Data are presented as mean ± SD. Sample: 24, n = 10; 28, n = 10; 32, n = 10; 36, n = 10.

## Data Availability

The original contributions presented in this study are included in the article. Further inquiries can be directed to the corresponding author.

## Abbreviations

The following abbreviations are used in this manuscript:

LD	Longissimus Dorsi Muscle
H&E	Hematoxylin and Eosin Staining
WBSF	Warner-Bratzler Shear Force
TEM	Transmission Electron Microscopy
IMF	Intramuscular Fat
SEM	Scanning Electron Microscope
HP	Hydroxylysine Pyridinoline
